# Etiology of Delayed Inflammatory Reaction Induced by Hyaluronic Acid Filler

**DOI:** 10.1055/a-2184-6554

**Published:** 2024-02-07

**Authors:** Won Lee, Sabrina Shah-Desai, Nark-Kyoung Rho, Jeongmok Cho

**Affiliations:** 1 Yonsei E1 Plastic Surgery Clinic, Scientific Faculty of the Minimal Invasive Plastic Surgery Association, Dongan-ro, Dongan-gu, Anyang, Republic of Korea; 2 Perfect Eyes Ltd, London, United Kingdom; 3 Department of Dermatology, Sungkyunkwan University School of Medicine, Gyeonggi-do, Republic of Korea. Leaders Aesthetic Laser & Cosmetic Surgery Center, Seoul, Republic of Korea; 4 Etonne Plastic Surgery Clinic, Scientific Faculty of the Milimal Invasive Plastic Surgery Association, Seoul, Republic of Korea

**Keywords:** hyaluronic acid filler, delayed inflammatory reaction, filler complication, delayed hypersensitivity

## Abstract

The etiology and pathophysiology of delayed inflammatory reactions caused by hyaluronic acid fillers have not yet been elucidated. Previous studies have suggested that the etiology can be attributed to the hyaluronic acid filler itself, patient's immunological status, infection, and injection technique. Hyaluronic acid fillers are composed of high-molecular weight hyaluronic acids that are chemically cross-linked using substances such as 1,4-butanediol diglycidyl ether (BDDE). The mechanism by which BDDE cross-links the two hyaluronic acid disaccharides is still unclear and it may exist as a fully reacted cross-linker, pendant cross-linker, deactivated cross-linker, and residual cross-linker. The hyaluronic acid filler also contains impurities such as silicone oil and aluminum during the manufacturing process. Impurities can induce a foreign body reaction when the hyaluronic acid filler is injected into the body. Aseptic hyaluronic acid filler injections should be performed while considering the possibility of biofilm formation or delayed inflammatory reaction. Delayed inflammatory reactions tend to occur when patients experience flu-like illnesses; thus, the patient's immunological status plays an important role in delayed inflammatory reactions. Large-bolus hyaluronic acid filler injections can induce foreign body reactions and carry a relatively high risk of granuloma formation.

## Introduction

Hyaluronic acid filler injections are a widely used minimally invasive aesthetic technique. 1 Although these injections are relatively low-risk procedure, they can cause significant adverse vascular and nonvascular complications. 2 Among the nonvascular complications, delayed inflammatory reactions have been a serious problem that are characterized by swelling and areas of induration presenting at least 2 weeks after the filler injection. 3 However, the etiology and pathophysiology of delayed inflammatory reactions have not yet been fully elucidated. 4 5 Further, there is variation in the terminology between articles, such as “delayed-onset reaction,” 5 “delayed inflammatory reaction,” 4 6 “delayed type hypersensitivity reaction,” 4 “delayed-onset tissue nodule,” 7 “nonantibody-mediated edema,” 8 and so on. Additionally, various studies have proposed that delayed inflammatory reaction results from biofilm, nodule, and/or granuloma. 9 10 However, nodules and granulomas may represent the progressive phenomena of a delayed inflammatory reaction rather than the etiology. The authors discuss the etiology of delayed inflammatory reactions based on previous studies, including reviewing the role of hyaluronic acid filler itself, patient's immunological status, infection, and injection technique.

## The Hyaluronic Acid Filler

Hyaluronic acid fillers are composed of high-molecular weight (MW) hyaluronic acids chemically cross-linked using substances such as 1,4-butanediol diglycidyl ether (BDDE). 11 Different products in the market, such as Nonanimal-Stabilized hyaluronic acid (HA), Cohesive Polydensified Matrix (CPM), Resilient HA, Safe Transparent Optimized Reliable Manufacturing, and Vycross Technology, have different manufacturing processes. 12 However, these processes usually involve reactions with BDDE, washing, and autoclaving. Although the manufacturing processes are similar, the final products differ significantly depending on the cross-linking time, temperature, and hyaluronic acid concentration. 13 Therefore, different products can induce different tissue reactions in humans, some of which are described below.

### 1,4-Butanediol Diglycidyl Ether

As previously described, hyaluronic acid fillers are composed of hyaluronic acids linked using a cross-linker, with the modification degree describing the percentage of hyaluronic acid disaccharide monomer units bound to a cross-linker molecule. 14 However, BDDE does not cross-link two hyaluronic acid disaccharides properly and they exist as fully reacted cross-linkers, pendant cross-linkers, deactivated cross-linkers, and residual cross-linkers. 11 Deactivated type is BDDE that has nonreacted HA but hydrolyzed form and residual type is native form. The Food and Drug Administration recommends a residual level of unreacted BDDE level of <2 ppm for safety. Therefore, unreacted BDDE, similar to residual BDDE, usually does not exist in high amounts in the final hyaluronic acid product. However, problems remain with the pendant and deactivated types. The deactivated cross-linker, 1,4-butanediol di-(propan-2,3-diolyl) ether (BDPE), is known for its major impurities. 15 In addition, there is a much higher proportion of the pendant type than the fully reacted type in hyaluronic acid fillers. 16 To describe these hyaluronic acid filler modifications, previous studies have proposed terms that can characterize hyaluronic acid hydrogels cross-linked with BDDE. 17 18

1. The degree of modification (MoD) is the stoichiometric ratio of the sum of mono- (pendant type) and double-linked (fully reacted type) BDPE residues and hyaluronic acid disaccharide units. MoD percentage increases with the increase in cross-link modifications seen when compared with the acetyl group.
2. The cross-linker ratio indicates the fraction of double-linked cross-linker residues compared with all linked cross-linkers and represents a measure of cross-linker efficiency.
3. The degree of cross-linking (CrD) is the stoichiometric ratio of double-linked BDPE residues and hyaluronic acid disaccharide units.

**Fig. 1:**
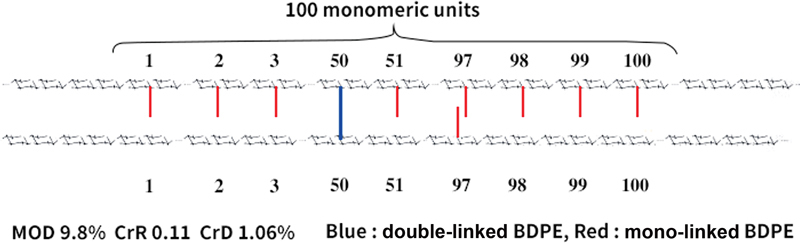
Schematic diagram of degree of cross-linking. Total degree of modification is 9.8% (schematically 10%) and degree of cross-linking is 1.06% (schematically 1%). BDPE, 1,4-butanediol di-(propan-2,3-diolyl) ether; CrD, degree of cross-linking; CrR, cross-linker ratio; MoD, degree of modification.

According to a previous study, CPM-2 had 9.8% MoD and 1.06% CrD, 19 as shown in Fig. 1 .

There is no evidence that pendant BDDE and deactivated BDDE (BDPE) are linked to delayed inflammatory reactions; however, the purity of an ideal hyaluronic acid filler product should be as high as possible for safety.

### Impurities Inside Hyaluronic Acid Filler

Our previous study revealed the presence of impure particles in hyaluronic acid filler products. 19 Stainless steel is commonly found in the machinery during manufacturing. 20 Aluminum particles may exist during the manufacture of prefilled glass syringes. 21 Aluminum can induce an enhanced humoral immune response. 22 Silicone oil, which is used as a lubricant in prefilled syringes, can also be detected inside the hyaluronic acid filler 19 and form particles. 23 Silicone oil can act as an adjuvant and promote immunological tolerance and induce an antibody response. 24 Thus, silicone oil may augment the delayed inflammatory response caused by hyaluronic acid fillers. 24 According to the United States Pharmacopeia 26 and the European Pharmacopeia, 27 the threshold for particle levels in prefilled syringes are 6,000 and 600 per container for particles ≥10 and ≥25 μm, respectively. That means particles which are bigger than 10 μm should not exceed 6,000 inside 1 mL of prefilled syringe. But there are more particles detected in 1 mL of HA filler products according to a previous study. 19 Although there is no direct evidence that impurities inside the hyaluronic acid filler cause a delayed inflammatory reaction, the hyaluronic acid filler product should be as pure as possible and not contain a large amount of impurities ( Table 1 ).

**Table 1:** Hyaluronic acid filler products with variable counts of insoluble particles 19

	Larger than 10 µm (count/syringe)				Larger than 25 µm (count/syringe)			
	First	Second	Third	Average	First	Second	Third	Average
Lorient No 6	1,204	1,340	1,196	1,246	88	72	88	82
A	76,520	79,792	76,588	77,633	812	1,044	876	910
B	11,468	10,460	10,804	10,910	312	312	236	286
C	16,264	16,816	17,084	16,721	936	916	936	929
D	121,244	120,184	118,140	119,856	6,156	6,600	6,008	6,254
E	27,912	29,712	28,180	28,601	120	84	96	100
F	28,892	25,780	25,480	26,717	896	756	820	824
G	46,408	43,516	40,568	43,497	992	1,100	1,092	1,061
Control	44	32	36	37	20	4	12	12

### Molecular Weight of Hyaluronic Acid

High-MW hyaluronic acid has weight greater than 1,000 kDa 7 and is known to inhibit inflammation because CD44 receptors produce anti-inflammatory cytokines. 27 28 The MW of hyaluronic acid used in the production of soft tissue fillers ranges from 500 to 6,000 kDa. The sodium salt of hyaluronan often occurs as a disaccharide with an MW of approximately 401 Da. 29 Hyaluronic acid fragments below 1,000 kDa are proinflammatory and can initiate an inflammatory response by activating Toll-like receptors 2 and 4. 30 Some studies have suggested that the Vycross technology hyaluronic acid filler may have a higher risk of delayed inflammatory reactions because of its low-MW hyaluronic acid composition. 31 However, although hyaluronic acid fillers usually contain a high-MW hyaluronic acid, it is degraded by hyaluronidase and reactive oxygen into 20-kDa fragments. 7 Thus, it is reasonable to assume that the periphery of the implanted hyaluronic acid filler can be degraded by hyaluronidase and that low-MW fragments of hyaluronic acid could induce an inflammatory response. However, the low-MW hyaluronic acid-induced inflammatory response is closely related to infection and the patient's immune status. 7 Moreover, the MW appears to have no impact on the inflammatory or immune response to fillers, regardless of hyaluronic acid cross-linking. 32 Further evaluation is required to determine the influence of MW on delayed inflammatory reactions.

### Manufacturing Process

**Fig. 2:**
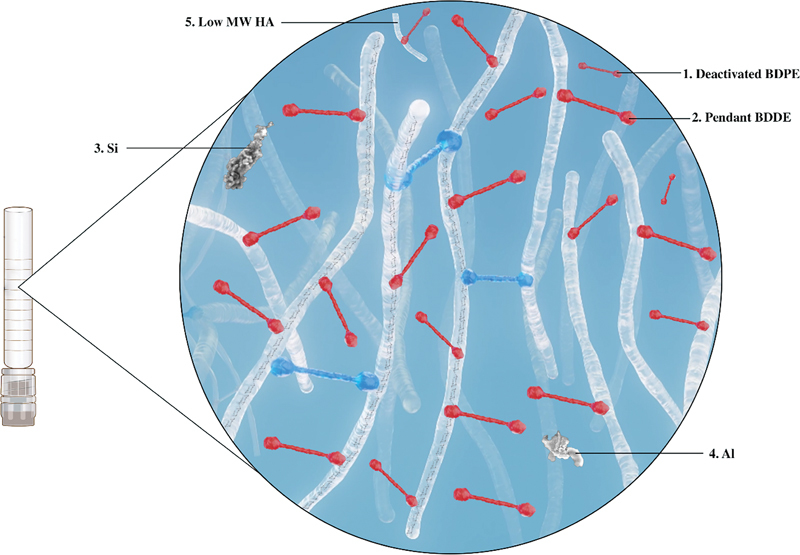
Possible hyaluronic acid filler impurities. BDPE, 1,4-butanediol di-(propan-2,3-diolyl) ether; MW, molecular weight.

In addition to BDDE, impurities, and low-MW hyaluronic acid, various substances can be present in the hyaluronic acid filler product. One such substance is raw hyaluronic acid. Hyaluronic acid fillers are usually derived from raw hyaluronic acid powder, 34 which is derived from bacteria, but its purity varies. 34 Different hyaluronic acid fillers can be used to produce different purities. In addition, because hyaluronic acid is produced from fermented streptococcal species, there may be some endotoxins present in the hyaluronic acid filler products. Therefore, the hyaluronic acid filler product should be purified such that the endotoxin concentration is <20 units per syringe. 36 During the manufacturing process, sodium hydroxide is used to create an ether linkage in the hydroxyl chain. 37 Thus, this highly alkaline solution should be removed during washing. There is no evidence that impurities induce delayed inflammatory reactions; however, an ideal hyaluronic acid filler product should be as pure as possible ( Fig. 2 ).

## Patient's Immunological Status

Hyaluronic acid is a natural component of the human tissue. 14 Even if hyaluronic acid is produced using bacteria, as is the case for most fillers, the hyaluronic acid molecule is identical and independent of the species and will not be recognized as a foreign material when implanted in the body. During the cross-linking process, it is important not to modify the hyaluronic acid molecule to such an extent that it is no longer recognized as hyaluronic acid, as this may lead to foreign body reactions. 37 The foreign body reaction is the final stage of inflammation and wound healing after implantation. 38 The purpose of a foreign body granulomatous reaction is to encapsulate and isolate foreign materials that cannot be removed immediately by enzymatic breakdown or phagocytosis. 10 However, the incidence of foreign body granuloma after hyaluronic acid filler injection has been reported to be 0.02 to 0.4%. 39 Therefore, after injection, the extent of foreign body reaction varies due to factors such as the hyaluronic acid itself, patient's immunological status, and injection volumes.

Additionally, the occurrence of nodules and granulomas cannot be used to predict which patients are at risk. 40 Delayed inflammatory reactions can occur without nodule formation. Therefore, in addition to nodule or granuloma formation, patient status is also important for delaying inflammatory reactions.

Delayed inflammatory reactions tend to occur in patients with flu-like illnesses. 41 Type IV hypersensitivity reactions initiated by T lymphocytes following hyaluronic acid injection and influenza infection may play a role in late-onset nodules. 41 However, a recent article reported that there was no T-cell activity in biopsies from areas with delayed inflammatory reactions. 42 Evidence suggests that viral and bacterial infections act as immunological trigger. 43 A recent study described that viremia and postvaccination status with a heightened immune status, and virulent bacteria seeding the surface of the filler in bacteremia, would likely induce a significant immune response. 7 Another study reported that patients with human leukocyte antigen subtypes B*08 and HLA subtype-DRB1*03 have an increased risk of delayed inflammatory reactions. 44

With the emergence of the coronavirus disease 2019 (COVID-19) pandemic caused by severe acute respiratory syndrome-related coronavirus 2 (SARS-CoV-2) virus, numerous vaccines have become available globally. 45 Reports of delayed inflammatory reactions to hyaluronic acid fillers have increased in after COVID-19 vaccination 46 and infections. 47 48 It has been suggested that the COVID-19 spike protein acts as a trigger for the formation of a delayed inflammatory reaction. 49 Spike protein interactions with angiotensin-converting enzyme receptors cause a proinflammatory helper T cell 1 response and promote CD8 ^+^ T cell-mediated reactions. 49 Anti-inflammatory drugs or steroids have been used for delayed inflammatory reaction. 13 48 50 However, because the COVID-19 vaccine is related to angiotensin-converting enzyme (ACE) 2 receptor, ACE inhibitors such as lisinopril have been proposed for the management of delayed inflammatory reactions. 51 Doses of 5 to 10 mg of lisinopril have been used, with early resolution of swelling within 24 hours in multiple cases. 52 Antihistamines are not beneficial for the management of delayed inflammatory reactions. 47 53 54 Thus, steroids or 10 mg lisinopril seem to be promising treatments for delayed inflammatory reactions associated with COVID-19 vaccines.

## Infection

The pathogenicity of any implanted surface bacteria affects the patient's immune response, and the patient is more likely to tolerate and implant normal skin commensal bacteria than true pathogens. 7 Thus, aseptic and clean practices should address recontamination during injection procedure. 43 To prevent infection, it is important to check patient's infection history, with previous filler injection history. It is also very important to remove patient's makeup completely before filler injection procedure. There is a risk of bacterial contamination with every needle passing through the skin; however, rapid degradation and phagocytosis may address the invading bacteria. 55 Chlorhexidine gluconate and isopropanol are the preferred antiseptic solutions. 43 Chlorhexidine use has been suggested to contribute to the very low incidence of infection in minimally invasive cosmetic procedures such as filler injections. 56 Thus, during hyaluronic acid filler injections, an aseptic environment should be maintained using an antiseptic solution.

## Bolus Injection

The association between a large volume of hyaluronic acid filler and delayed inflammatory reaction is controversial. 43 Larger boluses can cause mechanical irritation and trigger inflammatory reactions. 57 Ideal fillers should be nontoxic, biocompatible, reversible, and safe. 58 As previously described, even if hyaluronic acid is produced using bacteria, the hyaluronic acid molecule is identical and independent of the species and will not be recognized as a foreign material when implanted in the body. However, because the hyaluronic acid filler is cross-linked with a cross-linker, the filler can be recognized as a foreign body by the immune system. 40 Once the filler is recognized as a foreign body, phagocytosis occurs; however, this is related to the longevity of the filler. 59 Particles larger than 5 μm generally require aggregated macrophages (foreign body giant cells) to be phagocytosed, and particles larger than 15 to 20 μm are generally not ingested by macrophages or transported to the local lymph nodes. 60 The body's response varies with the composition of the filler; hyaluronic acid generates more lymphocytic infiltrate, while calcium hydroxyapatite generates more macrophages. 10 The intensity of the reaction depends on the immunological inertness of the injected material. 39 Before 1999, the reported rate of delayed inflammatory response to hyaluronic acid fillers was 0.7%. 61 With manufacturing improvements to increase the purity of hyaluronic acid products, the rate has decreased to approximately 0.2%. 10 However, impurities still exist, as described earlier, and even a large bolus injection increases the risk of foreign body reactions to form multinucleated giant cells. 62 63 When large amount of filler is injected, patient's immune response prolong for a longer time, which increases the possibility of biofilm formation. Thus, a large bolus volume of hyaluronic acid filler has a greater risk of causing foreign body reactions and other complications. 64 The term “tissue integration” refers to the “pattern of distribution within the biological tissue and, specifically, the way the filler material entangles itself in dermal fibers.” 65 Although there are differences between the layers and filler products, foreign body reactions do not occur or minimal cell infiltration occurs when the hyaluronic acid filler is properly integrated into the tissue. 66 Research in humans has also shown no inflammation or foreign body reaction when 0.2 mL of hyaluronic acid filler is injected intradermally. 67 Another study showed that the level of inflammatory reaction depends on the hyaluronic acid filler product. 68 Thus, when tissue integration is properly performed, no foreign body reaction occurs, and when there are no signs of inflammation, the hyaluronic acid filler degrades slowly within a year. 69

## Conclusion

The etiology of the delayed inflammatory reaction induced by hyaluronic acid fillers is uncertain. However delayed inflammatory reactions are related to foreign body reactions and recurrent inflammation around the injected hyaluronic acid filler. Thus, the injected filler should be as pure as possible without impurities. In addition, stringent aseptic techniques should be practised during hyaluronic acid filler injection.
